# Posterior Reversible Encephalopathy Syndrome: Tips for Diagnosis and Treatment

**DOI:** 10.7759/cureus.14087

**Published:** 2021-03-24

**Authors:** Kabeer Ali, Abhinav Karan, Shivonne Biswah, Surujpal Teelucksingh, Nazim Boris Mohammed

**Affiliations:** 1 Internal Medicine, Eric Williams Medical Sciences Complex, Champs Fleurs, TTO; 2 Internal Medicine, Medical Associates Hospital, St. Joseph, TTO; 3 Endocrinology, Medical Associates Hospital, St. Joseph, TTO; 4 Nephrology, Eric Williams Medical Sciences Complex, Champs Fleurs, TTO

**Keywords:** posterior reversible encephalopathy syndrome (pres), nephrology, radiology, critical care

## Abstract

Often described as a clinico-radiological entity, posterior reversible encephalopathy syndrome (PRES) is being increasingly diagnosed nowadays. However, mystery still surrounds its exact etiology. Though there are no standardized diagnostic criteria for this syndrome, there is a consistent feature associated with it: brain vasogenic edema in combination with neurotoxicity. The nonspecific nature of this condition leaves room for the diagnosis to be overlooked, leading to delays in providing appropriate treatment and unfavorable patient outcomes. PRES is associated with a variety of medical conditions including hypertension, eclampsia, autoimmune conditions, renal failure, sepsis, and an immunocompromised state, such as that secondary to the use of immunosuppressive therapy, human immunodeficiency virus (HIV), and organ transplants. Treatment by a multidisciplinary team and prompt identification and reversal of the underlying cause can lead to beneficial outcomes, as in the case we present in this report.

## Introduction

Posterior reversible encephalopathy syndrome (PRES) is still a poorly understood condition, requiring a combination of specific clinical and radiological findings to arrive at a correct diagnosis. In this report, we present a case of a 35-year-old male with hypertension and end-stage renal disease (ESRD) on renal replacement therapy (RRT) who presented with a first-time generalized tonic-clonic seizure and severely altered mental status. We focus on highlighting the aspects of diagnosis and treatment to guide and alert busy clinicians in the emergency room or ward regarding this potentially dangerous but eminently treatable condition.

## Case presentation

A 35-year-old male with ESRD secondary to a prolonged non-steroidal anti-inflammatory drug (NSAID) overuse and hypertension presented to the Accident and Emergency department of our hospital with a first-time generalized tonic-clonic seizure. The event had been witnessed by his seven- and 11-year-old sons, who had seen their father suddenly fall on the floor and begin to shake uncontrollably with stiffening of all limbs. The patient denied any history of headaches or visual changes prior to this incident. His last hemodialysis session had been on the day prior to the presentation, with a pre-dialysis urea level of 34 mg/dL and creatinine of 10.6 mg/dL. His post-dialysis urea was 15 mg/dL and his creatinine level was 6.1 mg/dL. He had been fully compliant with his medication regime of sevelamer carbonate, nifedipine, carvedilol, and furosemide, and his hypertension had been medically managed well on this therapy.

**Figure 1:**
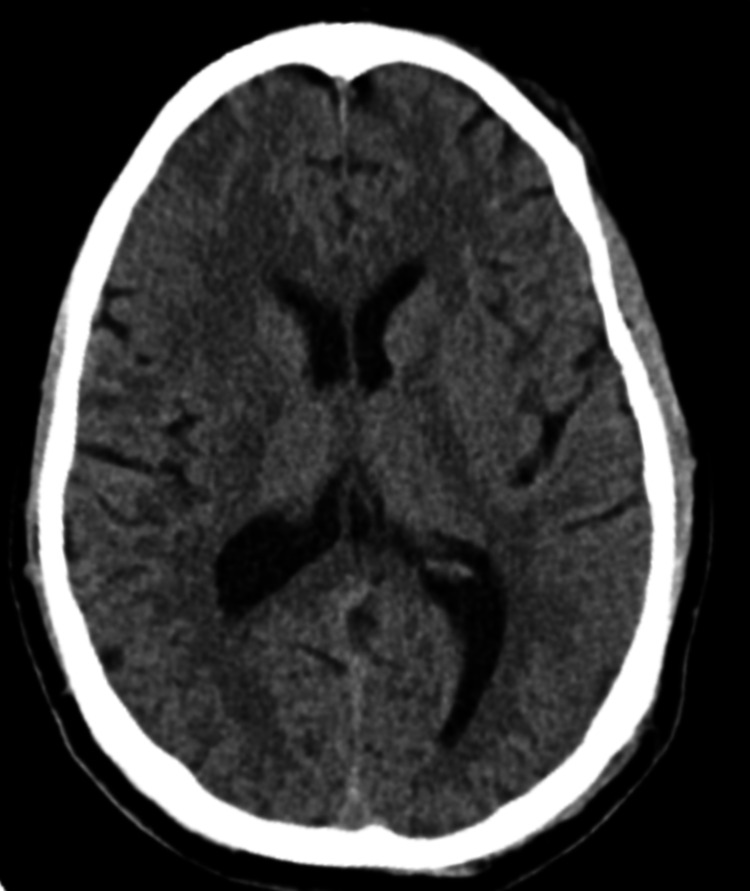
Axial view of the non-contrast CT scan of the brain The image showed no acute intracranial findings CT: computed tomography

On arrival to the Accident and Emergency department, 30 minutes after undergoing the ictus, the patient was still experiencing jerky movements of his extremities; he was also severely disoriented and combative. Initial vital signs were as follows: blood pressure of 212/139 mmHg, a heart rate of 72 beats per minute, blood sugar level of 143 mg/dL, temperature of 36.3 °C, and oxygen saturation of 94%. Physical examination revealed a Glasgow Coma Scale (GCS) score of 9/15 (2/4 for “eyes”, 2/5 for “verbal”, and 5/6 for “motor”). His pupils were non-dilated and equal and reactive to light. Limited fundoscopy performed in the Emergency department was unremarkable. The remainder of his physical examination revealed no abnormalities. The patient’s airway was stabilized, intravenous access was obtained, and an infusion of phenytoin 1 g over one hour and three doses of midazolam 5 mg were administered. He was promptly taken for a non-contrast CT scan of his brain, which revealed no acute intracranial pathology (Figure 1).

The patient's initial laboratory investigations revealed good oxygenation with a partial pressure of oxygen (PaO_2_) of 95 mmHg, but a severe respiratory acidosis with a mild leukocytosis with granulocytosis (Table 1). His family denied any prior aggressive behavior or personality changes, and the patient’s urine toxicology screen for marijuana and cocaine was negative.

**Table 1:** Laboratory values at presentation

Labs	Values	Reference range
White blood cell count	13.6	4–11 x 10^9^/L
Granulocyte count	76.4	50–70%
Hemoglobin	12.3	13–18 g/dL
Mean corpuscular volume	82.3	80–95 fL
Platelet	186	150–140 x 10^9^/L
Sodium	144	135–145 mEq/L
Potassium	5.4	3.5–5.5 mEq/L
Chloride	103	96–106 mEq/L
Blood urea nitrogen	16	7–20 mg/dL
Creatinine	6.4 (baseline: 6.1)	0.84–1.21 mg/dL
pH	6.986	7.35–7.45
PCO_2_	90.5	35–45 mEq/L
HCO_3_	21.6	22–28 mEq/L
Glucose	130	80–140 mg/dl
C-reactive protein	7.7	<10 mg/dL
Total bilirubin	1.5	<1.2 mg/dL

The patient was admitted to the Nephrology service and started on 4 mg dexamethasone thrice daily, diazepam 10 mg every six hours, phenytoin 100 mg thrice daily, as well as ceftriaxone 2 g twice daily, vancomycin 500 mg once daily, fluconazole 150 mg once daily, and acyclovir 400 mg once daily. His blood pressure remained persistently elevated at >188/126 mmHg, requiring the infusion of glyceryl trinitrate 50 mg in 50 ml normal saline titrated at 5 ml per hour, and hydralazine 10 mg every six hours. Lumbar puncture revealed clear occasional lymphocytes, polymorphs and red blood cells, normal Gram stain, and minimally elevated protein of 95 mg/dl (reference range: 15-60 mg/dl) and glucose of 84 mg/dl (reference range: 50-80 mg/dl) with a concomitant blood glucose level of 106 mg/dl.

The patient remained obtunded over the next several days. His blood culture revealed the presence of coagulase-negative *Staphylococcus aureus*, and hence imipenem 500 mg once daily was initiated, to which it was sensitive. Despite back-to-back hemodialysis sessions and initiation of anti-hypertensives, the patient’s blood pressure remained high at >180/120 mmHg, but it responded to the addition of a labetalol infusion of 300 mg in 100 ml normal saline, started at 25 ml per hour.

**Figure 2:**
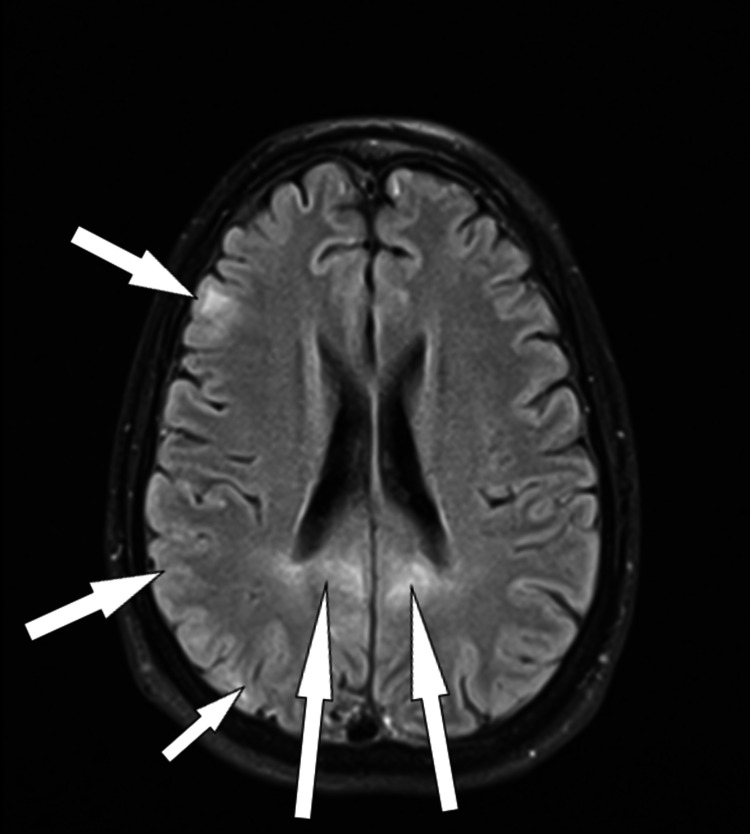
Axial view of FLAIR MRI The arrows signal parasagittal gyriform hyperintensity with bilateral distribution, consistent with PRES FLAIR: fluid-attenuated inversion recovery; MRI: magnetic resonance imaging; PRES: posterior reversible encephalopathy syndrome

**Figure 3:**
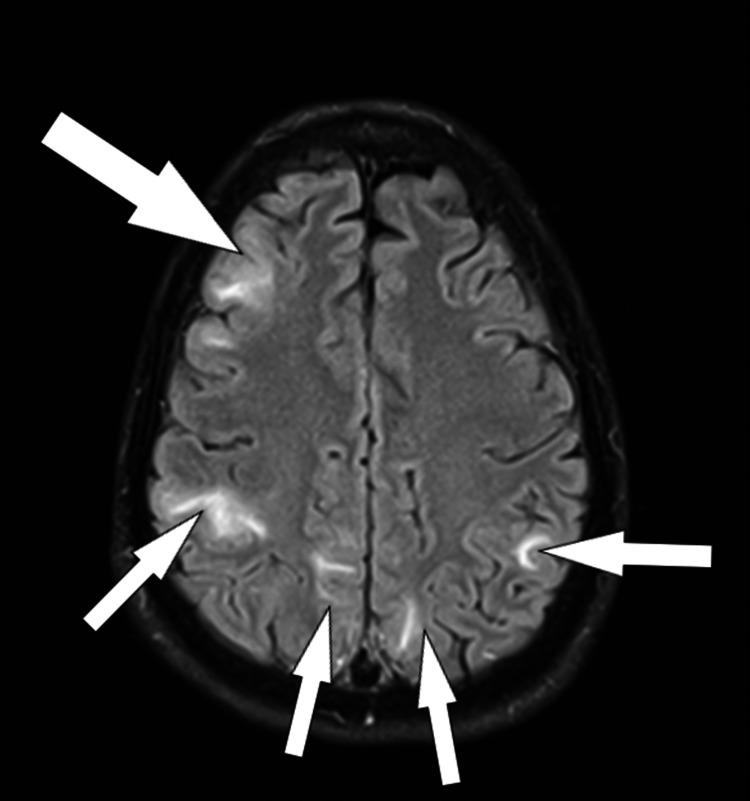
Another section of the same axial FLAIR MRI The arrows signal parasagittal gyriform hyperintensity with bilateral distribution FLAIR: fluid-attenuated inversion recovery; MRI: magnetic resonance imaging

Three days after his admission, the Neurology and Adult Intensive Care Unit (AICU) services were consulted; an electroencephalogram (EEG) was performed, and though the results were within normal limits, the decision was made by AICU to electively intubate the patient to protect his airway due to his fluctuating GCS, which varied from 8-11/15. An MRI of the patient’s brain was ordered, which revealed a parasagittal gyriform high T2/fluid-attenuated inversion recovery (FLAIR) signal with bilateral distribution consistent with PRES (Figures 2, 3).

The week after his admission, the patient’s blood pressure eventually returned to normal levels (systolic: 120-130; diastolic: 70-80), and he was weaned off the labetalol infusion. Daily chest and limb physiotherapy was performed. His GCS returned to the level of 15/15, and he was extubated and discharged from the AICU. As he improved clinically, his only reported symptom was a mild, persistent generalized headache. He received regular hemodialysis and had no further seizures apart from the initial one just before his presentation. Once he completed his course of antibiotics for his bloodstream infection, he was deemed stable enough to be discharged as he was found to be asymptomatic, and his laboratory investigations and vitals were within normal limits (Table 2). He attended regular nephrology, neurology, and physiotherapy out-patient follow-ups and has not had any recurrences of this presentation.

**Table 2:** Laboratory values at discharge

Labs	Value	Reference range
White blood cell count	4.7	4–11 x 10^9^/L
Hemoglobin	10.8	13–18 g/dL
Platelet	246	150–400 x 10^9^/L
Sodium	137	135–145 mEq/L
Potassium	4.2	3.5–5.5 mEq/L
Chloride	101	96–106 mEq/L
Blood urea nitrogen	12	7–20 mg/dL
Creatinine	2.95	0.84–1.21 mg/dL

## Discussion

Hinchey et al. first identified PRES in a case series of 15 patients, where 12 out of 15 (80%) had abrupt increases in blood pressure and eight out of 15 (53%) had impairment in renal function [1]. Both these independent risk factors were seen in our case as well: our patient was on hemodialysis with end-stage renal failure and was suffering from an acute hypertensive emergency (his initial BP was 212/139 mmHg). PRES has also been identified in several other conditions such as eclampsia, those on immunosuppressive therapy after transplantation, sepsis, autoimmune conditions such as systemic lupus erythematosus, porphyria, and it has even been recently reported in a case of coronavirus disease 2019 (COVID-19) [2].

Being a radiological diagnosis in part, the characteristic imaging findings of PRES include regions of focal symmetric hemispheric edema, commonly manifesting as hyperintensity affecting the parietal and occipital lobes; the frontal lobes, inferior temporal-occipital junction, and cerebellum may also be affected, indicating that PRES is not wholly a “posterior” occurrence. This is best seen on T2-FLAIR MRI [3].

The exact mechanism behind this edema remains incompletely understood, with two broad categories of theories put forward: the “hyperperfusion” theory and the “hypoperfusion” theory [4]. The hyperperfusion theory is more popular; it states that the edema is a result of a failure of the autoregulatory mechanism of cerebral blood flow. This results in a high systemic blood pressure leading to hyperperfusion, arteriolar dilatation, injury to the capillary bed, and vasogenic edema. However, according to this theory, BP is not elevated in 20-40% of cases of PRES, and the extent of edema does not always correlate with the severity of hypertension [3]. The hypoperfusion theory, on the other hand, proposes that endothelial cell dysfunction, which is seen in sepsis, immunocompromised states, and autoimmune diseases, leads to altered vascular tone and vasoconstriction. The resulting hypoxia leads to the secretion of vascular endothelial growth factor (VEGF) from cerebral vessels, which increases permeability and hence causes edema [5]. These different theories need not be mutually exclusive, and research is ongoing to better elucidate the exact pathophysiological mechanism involved.

Clinically, patients’ symptoms are on a wide spectrum of neurotoxicities such as headache, visual changes, paresis, hemianopsia, and altered mentation. In this case, our patient presented with a first-time generalized tonic seizure, which happens to be a common presentation of PRES (seen in up to 66% of patients according to one retrospective study) [6]. Other neurotoxic symptoms exhibited by our patient include an altered mental state and severe persistent headache.

A diagnostic criterion put forward by Fugate et al. proposes that the presence of more than one acute neurological symptom coupled with more than one risk factor (e.g., severe hypertension or renal failure with suggestive MRI findings) warrants the diagnosis of PRES provided that there is no alternative diagnosis [7].

Based on these guidelines, our patient met the requirements to be diagnosed with PRES and was treated accordingly. Other causes of acute delirium were ruled out: cerebrospinal fluid (CSF) analysis was obtained to rule out an infectious or inflammatory process, and urine toxicology for marijuana and cocaine screening was negative. The patient did not consume alcohol, and liver function tests were within normal limits, excluding the possibility of hepatic encephalopathy. He had also undergone dialysis the day prior to the presentation, ruling out uremic encephalopathy.

The mechanism by which altered renal function contributes to the development of PRES is multifactorial. Renal failure is associated with marked hypertension, which is linked to extracellular volume expansion. Azotemia and electrolyte imbalances may cause interstitial brain edema by increasing the permeability of capillaries and cytotoxic edema by direct injury of the brain parenchyma [8]. Measures to maintain fluid equilibrium include a fluid and salt-restricted diet, use of appropriate medications such as loop diuretics and angiotensin-converting enzyme (ACE) inhibitors (once not contraindicated), and regular RRT.

Dialysis-dependent renal failure in itself categorizes a patient as being immunocompromised, even in the absence of uremia. Disorders of the pattern-recognition receptors system in renal disease patients result in impaired function of cells engaged in innate immunity. For example, monocytes and monocyte-derived dendritic cells have been shown to display decreased endocytosis and impaired maturation in ESRD patients [9]. Another study has reported that neutrophil bactericidal capabilities are reduced in hemodialysis patients [10]. It is not imprudent to infer that the main risk factors associated with the development of PRES in this instance were a combination of uncontrolled hypertension and the patient's immunocompromised state complicated by sepsis, both consequences of renal failure.

Interestingly, pre-existing brain lesions may predispose patients on hemodialysis to PRES. White matter lesions are common in individuals with kidney disease and are probably associated with vascular disease. Both baseline and post-dialysis images of such patients show increased apparent diffusion coefficient (ADC), indicative of interstitial edema [4].

It is also essential that the diagnosis of PRES is carefully distinguished from its mimickers. PRES can frequently be mistaken for other encephalitis syndromes. Of note, reversible cerebral vasoconstriction syndrome (RCVS) is a similar clinico-radiological entity commonly mistaken for PRES. It is in fact believed that RCVS and PRES lie on a spectrum of diseases that cause reversible, posterior-predominant cerebral edema, and they share many pathological mechanisms. PRES should be diagnosed when there is (1) a clinical history of acute/subacute neurologic changes including seizure, headache, encephalopathy, visual disturbance, or focal deficit, (2) cerebral imaging revealing focal vasogenic edema, and (3) clinical or radiologic proof of reversibility. RCVS can be diagnosed based on the evidence of reversible vasospasm involving cerebral arteries in the absence of other diagnoses that better account for vasoconstriction. In either case, prompt recognition and management are essential for a satisfactory clinical outcome [11].

Differentiation should also be made from dialysis disequilibration syndrome (DDS), particularly in patients on dialysis such as ours. In our case, DDS was low on our list of differential diagnoses as it is most commonly seen in patients on their first session of dialysis within 24 hours of their last dialysis. Furthermore, while imaging findings in PRES reveal cerebral edema predominantly localized to the posterior cerebral lobes, cerebral edema in DDS can be much more diffuse.

Prompt treatment of PRES is associated with a positive prognosis in the majority of cases. However, it can be argued that the term “reversible” in PRES is a misnomer, as mortality has been shown to be as high as 19%, mostly from intracranial hemorrhage and permanent neurologic sequelae [11]. The treatment essentially involves addressing underlying causes, which can be difficult if the underlying etiology is uncertain. Delays in treatment may result in irreversible damage leading to death or increased morbidity in the form of prolonged rehabilitation. A multidisciplinary, multispecialty approach should be implemented for this condition from the time of admission, which should continue post-discharge.

The diagnosis of PRES in our case was reinforced by the improvement of symptoms after the initiation of antihypertensives. It is recommended to exercise caution when lowering the blood pressure more slowly to reduce the risk of secondary ischemia and possibly infarction [12]. Intravenous antihypertensive medications including hydralazine, labetalol, and sodium nitroprusside are commonly used. Electrolyte disturbances and fluid overload should be corrected. Seizures may progress to status epilepticus and may require intravenous anticonvulsants for treatment. If applicable, reducing dosages of immunosuppressive medications may provide benefit, but this needs to be balanced with the risk of disease exacerbation and graft rejection in transplant recipients [13].

Follow-up MRI is not routinely recommended unless there is no complete resolution of symptoms; however, close ambulatory BP monitoring is recommended post-discharge, and cases of recurrent PRES have been described in the literature [14]. Multidisciplinary care should continue well into the outpatient period.

PRES is a nebulous condition that is frequently missed by physicians. Further research is necessary to gain more information on this condition. In particular, a possible avenue for investigation can be the use of corticosteroids. While corticosteroids are known to improve vasogenic edema, their use in PRES is of unknown significance. Furthermore, the long-term impact of PRES must also be investigated, as, despite having the misnomer “reversible” in its name, long-term complications arise in as much as 15% of patients [15].

PRES is a frequently misdiagnosed condition requiring a high index of suspicion, diagnostic acumen, and effective treatment strategies. Further research is required to devise optimal management methods for PRES in certain subgroups of patients and on the relative efficacy of treatment modalities.

## Conclusions

We presented a case of a 35-year-old male patient with a first-time generalized tonic-clonic seizure, leading to an eventual diagnosis of PRES in a setting of hypertension, ESRD, and bloodstream infection. Early imaging with MRI and maintaining a high clinical suspicion are crucial for making a diagnosis of PRES. Prompt and early treatment targeting the underlying cause should be initiated to achieve favorable outcomes, thereby making this syndrome a truly “reversible” entity. Early multidisciplinary involvement also reduces long-term morbidity and mortality.
